# Tracing the path from preschool wheezing to asthma

**DOI:** 10.1002/ppul.27305

**Published:** 2024-10-03

**Authors:** Ellen Kong, Darije Custovic, Adnan Custovic

**Affiliations:** ^1^ National Heart and Lung Institute Imperial College London London UK

**Keywords:** machine learning, pediatric asthma, wheezing phenotypes

## Abstract

This short review illustrates, using two recent studies, the potential and challenges of using machine learning methods to identify phenotypes of wheezing and asthma from childhood onwards.

Wheezing in preschool children is common, with half experiencing at least one episode before their sixth birthday, but it is also highly heterogeneous. The progression of preschool wheeze into either later childhood asthma or into symptom resolution commences from near‐identical clinical presentation in early life, though the pathophysiology underlying these differences remains obscure.^1^ Although “not all wheeze is asthma,” the evolution of preschool wheeze into later asthma is of particular interest. However, to further complicate matters, asthma is now recognized as an aggregate diagnosis comprising several mostly unknown endotypes, each perhaps being a mix of common (overlapping) and distinct mechanisms.^2^

Tracing the path—or paths—from preschool wheeze to later asthma therefore requires the proper identification and characterization of these endotypes. One approach to this focuses on using unsupervised machine learning methods applied to data about wheeze across time; the aim is to identify latent (hidden) patterns in the mass of data, grouping together participants who exhibit similar temporal patterns of wheeze into so‐called “phenotypes” which, it is assumed, might reflect different clusters of underlying mechanisms.^3^ Here, we illustrate the successes of and challenges with this approach using two recent analyses of the same data drawing from the five birth cohorts, and provide outline suggestions for future research.

In the first, Oksel et al.^1^ used binary latent class analysis (LCA) to tease out five distinct wheeze phenotypes from 7719 children with complete data on wheeze presence at five time‐points from a cross‐cohort harmonized data set. These phenotypes were labeled never/infrequent (52.1%), early‐onset preschool‐remitting (23.9%), early‐onset mid‐childhood‐remitting (9%), persistent (7.9%), and late‐onset wheeze (7.1%). Compared to never/infrequent wheeze, all phenotypes had higher odds of having asthma diagnosis by adolescence, with the association unsurprisingly being stronger for persistent wheeze and late‐onset wheeze. Similarly, the strongest association with allergic senitization in school‐age was observed for persistent wheeze; the two early‐onset remitting classes differed substantially in their association with senitization, with little evidence of association for preschool‐remitting, but a clear association for mid‐childhood‐remitting wheeze. The wheezing phenotypes were also differentially associated with risk factors such as maternal smoking and maternal asthma.

These differing associations per phenotype with various outcomes suggest that the derived classes have some sort of biological significance; however, closer inspection raises some issues. First, there is considerable heterogeneity within groups, with individuals with varying wheeze patterns being assigned to the same group (particularly in late‐onset wheeze); conversely, participants with identical wheeze patterns were sometimes assigned to different phenotypes. Relatedly, class labels do not always reflect individual wheezing patterns: most notably, some children assigned as early‐onset preschool‐remitting reported wheezing at later time points. Second, there was some instability in individual allocation to groups, with 12% of children having low membership probability (<.60) to any phenotype. Class membership certainty was highest in the persistent and never/infrequent classes, and lowest in late‐onset wheeze (with 51% of participants having membership probabilities <.80). Notably, these membership probabilities shifted substantially when missing data were imputed, with 23% of children changing wheeze phenotypes post‐imputation.

To address these problems with homogeneity and stability, Haider et al.^4^ re‐analyzed the same data set with a nonbinary partition‐around‐medoids approach, using six variables of wheezing spells related to time and frequency at each time‐point (rather than just presence). This approach found five *different* wheeze clusters: never, early transient, late‐onset, intermittent, and persistent wheeze. These clusters were more stable, internally homogeneous, and less affected by data imputation strategies. Once again, compared to the nonwheezing group, the remaining phenotypes had a stronger association with asthma diagnosis; persistent, late‐onset, and intermittent types had strong associations, while early transient wheezing was only weakly associated. Further, persistent and intermittent wheezers had significantly worse lung function and higher chances of having polymorphisms at 17q12‐21 and *CDHR3*, known asthma‐related loci.^4^ This analysis shows the potential of integrating “omics” data with clinical datasets, and that by leveraging advanced statistical models, we can gain insight into underpinning pathophysiology.

Both studies, then, were able to identify plausibly meaningful subtypes of childhood wheezing possessing significant associations to risk factors and outcomes, and even genetically correlates—however, the subtypes identified were different. This discrepancy is representative of the phenotyping approach more broadly: the wheeze phenotypes identified in such studies, while often labeled identically, differ significantly in their characteristics and associated risk factors.^5^ These variations are attributable at least in part to differences in study populations and designs, operational definitions, and analytical approaches.^5^ Unfortunately, such deviations in phenotypic characteristics may hinder attempts at more in‐depth explorations of wheeze‐inducing pathophysiology and limit the accuracy of risk prediction in at‐risk children. Ultimately, it is important to remember that purported wheeze phenotypes may or may not be an accurate reflection of underlying pathophysiological mechanisms: these phenotypic clusters are only *theoretical suggestions until proven otherwise* through rigorous validation in mechanistic studies and across other cohorts, in combination with scrutiny from clinical, biological, and genetics experts.^3^

Nonetheless, phenotyping studies have shown that there are identifiable subtypes of preschool wheezing with varying associations to later asthma diagnosis, allergic senitization, and genetic and environmental factors, vindicating the idea that there are numerous paths from preschool wheezing to later “asthmas.” Fully resolving these paths will require building on existing research in at least three ways. First, it will be important not to overlook the insights that have been gained through epidemiological and mechanistic studies into the mechanisms underlying asthma.^2^ The studies outlined above used only wheezing history to determine phenotypes; it is possible that incorporating other symptoms and the knowledge of the effects of lower‐airway pathogens, allergic senitization, immune system development, and gene–environment interactions gleaned from other studies into similar analyses may yield more accurate phenotypes. Second, epidemiological, genetic, and mechanistic studies might use purported phenotypes to provide more precise targets than a generalized notion of “asthma diagnosis.” Finally, we must acknowledge that at present there is no singular “best” analytical method for exploring wheeze‐asthma trajectories, and that the key to advancing our understanding may lie in employing a semi‐supervised approach that maximizes the strengths of supervised and unsupervised learning models.^3^ All these suggestions require multidisciplinary collaboration to draw together diverse expertise, which combined could enable us to better understand the various progression pathways and biological mechanisms of preschool wheezing.

## AUTHOR CONTRIBUTIONS

**Ellen Kong**: Writing—original draft; conceptualization. **Darije Custovic**: Writing—review and editing; conceptualization; supervision. **Adnan Custovic**: Writing—review and editing; supervision.

## CONFLICT OF INTEREST STATEMENT

Adnan Custovic reports personal fees from Novartis, personal fees from Sanofi, personal fees from Stallergenes Greer, personal fees from AstraZeneca, personal fees from GSK, and personal fees from La Roche‐Posay, outside the submitted work. The other authors declare no conflict of interest.

## Data Availability

This is a review article, the data statement does not apply.
